# Presentation, Prognostic Factors and Patterns of Failure in Adult Rhabdomyosarcoma

**DOI:** 10.1080/1357714031000114147

**Published:** 2003-03

**Authors:** James H. Simon, Arnold C. Paulino, Justine M. Ritchie, Nina A. Mayr, John M. Buatti

**Affiliations:** 1 Department of Radiation Oncology University of Iowa College of Medicine Atlanta GA 30322 USA; 2 Department of Pediatrics University of Iowa College of Medicine Atlanta GA USA; 3 Department of Biostatistics University of Iowa School of Public Health Iowa City IA 52242 USA; 4 Emory Clinic Department of Radiation Oncology 1365 Clifton Road NE Rm A1300 Atlanta GA 30322 USA

## Abstract

*Purpose*: The purpose of our study is to retrospectively review our institutional experience with adult rhabdomyosarcoma (RMS) to determine presentation, prognostic factors and patterns of failure in this disease.

*Materials and Methods*: All patients ≥ 16 years with a diagnosis of rhabdomyosarcoma were retrospectively reviewed. Tumors were classified according to the Intergroup Rhabdomyosarcoma Study (IRS) staging and grouping system. Median follow-up for surviving patients was 12.5 years.

*Results:* A total of 39 patients (23 male, 16 female) were seen at our institution from 1961 to 1999. Median age was 45 years, and age distribution showed a bimodal peak at the late teens to twenties and later in the sixties. Tumor in the extremity was most common as seen in 15 (39%); this was followed by head and neck in 11 (28%), genitourinary in eight (20%), trunk/retroperitoneum in four (10%) and other in one (3%). Tumor stage was T1 in 21 (52%) while 26 (67%) were > 5 cm in size. Pleomorphic histology was most common (36%) and increased in incidence according to age category: 0% for ages 16–19, 27% for ages 20–49 and 60% for ages ≥ 50 years old (*P* < 0.01). The median survival for the entire population was 2.25 years with a 5-year overall survival rate of 35%. Multivariate analysis identified early IRS stage (*P* < 0.001), non-embryonal histology (*P* < 0.009), favorable site (*P* = 0.024), female gender (*P* = 0.034), early T-stage (*P* = 0.034) and absence of nodal metastases (*P* = 0.037) as predictors of a better survival. The 5-year progression-free survival rate was 21%. Female gender (*P* = 0.002), non-embryonal histology (*P* = 0.009), early IRS stage (*P* = 0.02) and early T stage (*P* = 0.033) were found on multivariate analysis to predict for improved progression-free survival. The 5-year local control rate was 51%, and multivariate analysis found that only early T-stage was predictive of better local control (*P* = 0.045). Five of six Group II patients and five of eight Group III patients who received radiotherapy (RT) were locally controlled. Only one of five Group III patients was locally controlled without RT.

*Conclusions:* The overall prognosis of adult RMS is worse than reported in children, but age criteria within the adult population did not further classify outcome. RT is an effective local modality providing local control in almost all Group II and majority of Group III patients.


*Sarcoma* (2003) 7, 1–7

Taylor & Francis healthsciences

**ORIGINAL ARTICLE**

### Presentation, prognostic factors and patterns of failure in adult rhabdomyosarcoma*

JAMES H. SIMON[1], ARNOLD C. PAULINO[1,2], JUSTINE M. RITCHIE[3], NINA A. MAYR[1] & JOHN M. BUATTI[1]

[1]*Departments of Radiation Oncology and* [2]*Pediatrics, University of Iowa College of Medicine and* [3]*Department of Biostatistics, University of Iowa School of Public Health, Iowa City, IA 52242, USA*

**Abstract**

*Purpose*: The purpose of our study is to retrospectively review our institutional experience with adult rhabdomyosarcoma (RMS) to determine presentation, prognostic factors and patterns of failure in this disease.

*Materials and Methods*: All patients ≥ 16 years with a diagnosis of rhabdomyosarcoma were retrospectively reviewed. Tumors were classified according to the Intergroup Rhabdomyosarcoma Study (IRS) staging and grouping system. Median follow-up for surviving patients was 12.5 years.

*Results:* A total of 39 patients (23 male, 16 female) were seen at our institution from 1961 to 1999. Median age was 45 years, and age distribution showed a bimodal peak at the late teens to twenties and later in the sixties. Tumor in the extremity was most common as seen in 15 (39%); this was followed by head and neck in 11 (28%), genitourinary in eight (20%), trunk/retroperitoneum in four (10%) and other in one (3%). Tumor stage was T1 in 21 (52%) while 26 (67%) were > 5 cm in size. Pleomorphic histology was most common (36%) and increased in incidence according to age category: 0% for ages 16–19, 27% for ages 20–49 and 60% for ages ≥ 50 years old ($P < 0.01$). The median survival for the entire population was 2.25 years with a 5-year overall survival rate of 35%. Multivariate analysis identified early IRS stage ($P < 0.001$), non-embryonal histology ($P < 0.009$), favorable site ($P = 0.024$), female gender ($P = 0.034$), early T-stage ($P = 0.034$) and absence of nodal metastases ($P = 0.037$) as predictors of a better survival. The 5-year progression-free survival rate was 21%. Female gender ($P = 0.002$), non-embryonal histology ($P = 0.009$), early IRS stage ($P = 0.02$) and early T stage ($P = 0.033$) were found on multivariate analysis to predict for improved progression-free survival. The 5-year local control rate was 51%, and multivariate analysis found that only early T-stage was predictive of better local control ($P = 0.045$). Five of six Group II patients and five of eight Group III patients who received radiotherapy (RT) were locally controlled. Only one of five Group III patients was locally controlled without RT.

*Conclusions:* The overall prognosis of adult RMS is worse than reported in children, but age criteria within the adult population did not further classify outcome. RT is an effective local modality providing local control in almost all Group II and majority of Group III patients.

**Key Words:** *rhabdomyosarcoma, adults, extremity, radiotherapy*

### Introduction

Rhabdomyosarcoma (RMS) is the most common soft tissue sarcoma in the pediatric population. The median age at diagnosis is 5 years, and the majority of reported cases are in children. As a result of the cooperative efforts of the Intergroup Rhabdomyosarcoma Study (IRS) Committee, children now have expected 3-year overall and failure-free survival rates of 86 and 77%.[1] The IRS trials have identified effective chemotherapy regimens and have more clearly delineated the roles of surgery and radiotherapy (RT).

Adults are uncommonly afflicted with RMS. In a study from Memorial Sloan-Kettering Cancer Center, RMS comprised 2% of all soft tissue

Presented in part at the 2001 American Society of Therapeutic Radiology and Oncology meeting at San Francisco, CA (4–9 November 2001).

Correspondence to: Arnold C. Paulino, M.D., Emory Clinic, Department of Radiation Oncology, 1365 Clifton Road NE, Rm A1300, Atlanta, GA 30322, USA. Tel.: +1-404-778-5782. Fax: +1-404-778-5152. E-mail: arnold@radonc.emory.org

ISSN 1357-714X print/ISSN 1369–1643 online/03/010001-7 © 2003 Taylor & Francis Ltd
DOI: 10.1080/1357714031000114147

sarcoma in patients ≥ 16 years of age.[2] As a result, there is paucity of information regarding the behavior of this disease in this population. Few studies exist, with limited numbers of patients. The purpose of our study is to review our institutional experience with adult rhabdomyosarcoma in an effort to better define the presentation, prognostic factors and patterns of failure.

### Materials and methods

After obtaining internal review board approval, all patients with a diagnosis of rhabdomyosarcoma were identified in the tumor registry during the period 1961–1999. All available medical and radiotherapy records were reviewed. From this original cohort of patients, the study population ≥ 16 years of age at diagnosis was identified. Although the IRS included patients 16–21 years, we chose to study cases ≥ 16 years because most of the available literature in adults have included this age group. Pathologists at our institution reviewed all biopsies and surgical specimens. Immunohistochemical stains for desmin and muscle-specific actin were positive for all patients. In the latter part of the study, electron microscopy and/or anti-Myo D1 stains were also performed to further confirm the diagnosis of rhabdomyosarcoma. Based on the above tests, two patients originally thought to be malignant fibrous histiocytoma were reclassified as pleomorphic RMS. Cytogenetic studies were performed only in the last 9 years; abnormalities such as reciprocal translocations between chromosomes 2 and 13 or between 1 and 13 verified the diagnosis of alveolar rhabdomyosarcoma. In five patients, the RMS subtype could not be assessed as these cases were from the earlier part of the study, and materials for pathological review were not available during the time of this analysis.

Tumors were retrospectively classified according to the IRS pretreatment staging and grouping system and presented in Tables 1 and 2.[1] The following variables were extracted from the existing records: gender, age, *T* stage, *N* stage, *M* stage, size, location, histology and type of treatment. Tumors were classified according to sites found to carry prognostic significance in childhood: favorable (orbit, paratesticular, non-parameningeal head and neck) or unfavorable (extremity, bladder, prostate, parameningeal sites, retroperitoneum, trunk, other). Non-embryonal histology included those with pleomorphic, alveolar, mixed alveolar–pleomorphic, botryoides and NOS categories. Although sarcoma botryoides is a subtype of embryonal histology, we analyzed the classic embryonal histology as an entity because of the better prognosis of the botryoides variety in children. Thus for the rest of this study, embryonal refers to the classic embryonal histology and does not include the botryoides subtype. Surgery, RT and chemotherapy (CT) were employed alone or in varying combinations. The study endpoints were overall survival, progression-free survival and local control and were estimated using the Kaplan–Meier method.[3] Cox proportional hazards regression models were used to identify factors associated with time until the event of interest.[4] The backward stepwise model building technique was the multivariate analysis approach used to identify factors associated with time to event of interest. Statistical tests were performed using the SAS system.[5] The median follow-up for all patients and for surviving patients was 5.7 and 12.5 years, respectively.

**Table 2.** *Intergroup rhabdomyosarcoma study surgical grouping system*

| | |
|---|---|
| Group I | Localized disease, completely resected |
| Group II | Compromised or regional resection |
| A | Grossly resected with microscopic residual tumor |
| B | Regional disease, completely resected, with nodes involved and/or tumor extension into an adjacent organ |
| C | Regional disease with involved nodes, grossly resected, but with evidence of microscopic residual tumor |
| Group III | incomplete resection or biopsy with gross residual disease |
| Group IV | Distant disease at diagnosis |

**Table 1.** *Intergroup rhabdomyosarcoma pretreatment staging system*

| Stage | Site | Invasiveness | Size | Nodal status | Metastasis |
|---|---|---|---|---|---|
| Stage 1 | Favorable | T1 or T2 | a or b | N0 or N1 | M0 |
| Stage 2 | Unfavorable | T1 or T2 | a | N0 | M0 |
| Stage 3 | Unfavorable | T1 or T2 | b | N0 | M0 |
| | Unfavorable | T1 or T2 | a or b | N1 | M0 |
| Stage 4 | Favorable or Unfavorable | T1 or T2 | a or b | N0 or N1 | M1 |

Favorable sites: non-bladder/non-prostate genitourinary sites, orbit, non-parameningeal head and neck. Unfavorable sites: parameningeal head and neck, extremity, trunk, retroperitoneum. Invasiveness: T1, confined to site/organ of origin; T2, extension beyond site/organ of origin. Size: a, ≤ 5 cm; b, > 5 cm.

### Results

*Incidence and host characteristics*

A total of 39 patients (23 male, 16 female) $\geq 16$ years were identified which represented 30% of all 130 rhabdomyosarcoma cases seen at the University of Iowa during this time period. The median age at diagnosis was 45 years. The distribution of age at diagnosis is presented in Figure 1. A bimodal peak was seen in the late teens to twenties and fifties to seventies. Age distribution in years was as follows: 16–19 (26%), 20–29 (18%), 30–39 (2%), 40–49 (8%), 50–59 (13%), 60–69 (21%), 70+ (13%).

*Tumor characteristics*

The primary site location was the extremities in 15 (39%), head and neck in 11 (28%), genitourinary system in eight (20%), trunk/retroperitoneum in four (10%) and other in one (3%). The anatomic subtypes of tumor location are presented in Table 3. The orbit as a head and neck site was only found in one patient who was 17 years. The majority of tumors (52%) was T1 and did not invade other structures. Two-thirds were larger than 5 cm. The histological subtype was pleomorphic in 14 (36%), embryonal in 10 (26%), alveolar in eight (20%), mixed alveolar-pleomorphic in one (3%), sarcoma botryoides in one (3%) and not otherwise specified (NOS) in five (13%). The distribution of histological types according of age of patient is shown in Table 4.

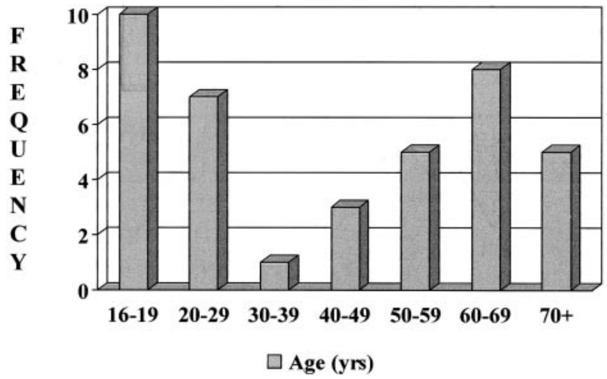

**Fig. 1.** *Distribution of rhabdomyosarcoma cases according to age.*

Pleomorphic histology was not found in any patients 16–19 years of age but was seen in 27 and 60% of those aged 20–49 and $\geq 50$ years, respectively ($P < 0.001$). On the other hand, embryonal histology was seen in 80, 18 and 0% of patients aged 16–19, 20–49 and $\geq 50$ years, respectively.

Regional nodal metastasis was noted in seven (18%), while distant metastasis was seen in six patients (15%). Of the seven who had regional nodal metastases at initial diagnosis, primary site location was the extremity in two, paratesticular region in two, soft palate in one, prostate in one and mediastinum in one. For the six who initially presented with distant metastasis, the sites of spread were pulmonary in three, pulmonary and bone in one, bone marrow in one and liver in one. IRS Stage distribution was as follows: Stage 1 in five (12%), Stage 2 in 11 (28%), Stage 3 in 17 (44%) and Stage 4 in six (15%). Group classification was I in 14 (36%), II in six (15%), III in 13 (33%) and IV in six (15%).

*Treatment*

Treatment varied over the study period. Twenty-eight patients (72%) had an attempted resection; subtotal resection with gross residual disease, total

**Table 3.** *Anatomic distribution of adult rhabdomyosarcoma*

| Location | Frequency |
|---|---|
| Extremity | 15 (38%) |
| Lower extremity | 11 |
| Upper extremity | 4 |
| Head and neck | 11 (28%) |
| Paranasal sinuses | 4 |
| Nasopharynx | 2 |
| Pterygopalatine Fossa | 1 |
| Buccal mucosa | 1 |
| Soft Palate | 1 |
| Neck | 1 |
| Orbit | 1 |
| Genitourinary | 8 (21%) |
| Paratesticular | 4 |
| Bladder | 2 |
| Prostate | 2 |
| Retroperitoneum | 2 (5%) |
| Trunk | 2 (5%) |
| Mediastinum | 1 (3%) |

**Table 4.** *Number of patients according to age category and histology*

| Histology | 16–19 years | 20–49 years | 50+ years | Total (%) |
|---|---|---|---|---|
| Embryonal | 8 | 2 | 0 | 10 (26) |
| Alveolar | 2 | 3 | 3 | 8 (21) |
| Pleomorphic | 0 | 3 | 11 | 14 (36) |
| Botryoides | 0 | 0 | 1 | 1 (2) |
| Mixed Alveolar-pleomorphic | 0 | 1 | 0 | 1 (2) |
| NOS | 0 | 2 | 3 | 5 (13) |
| Total | 10 (26) | 11 (28) | 18 (46) | 39 (100) |

NOS, not otherwise specified.

resection with microscopic positive margins and total resection with microscopic negative margins were achieved in eight, six and 14 patients, respectively. The other 11 patients (28%) had a biopsy only. Chemotherapy was administered to 22 (56%) and was more likely to be used after 1980. The most commonly employed agents were vincristine (V), actinomycin-D (A) and cyclophosphamide in seven, actinomycin-D alone in four and VA in three patients. Radiotherapy (RT) was given to 20 patients. RT was delivered to three Group I, six Group II, eight Group III and three Group IV patients. Treatment was to the primary site unless there was pathologic nodal involvement; the draining lymphatics were treated with nodal disease. Median dose was 5040 cGy with a range from 4000 to 6660 cGy using 180–200-cGy fractions. One patient with orbital RMS received 5550 cGy using 110 cGy given in a hyperfractionated fashion.

*Overall and progression-free survival*

The median survival for the study population was 2.25 years. Five-year overall survival was 35% (Figure 2). On multivariate analysis, early IRS stage ($P = 0.001$), non-embryonal histology ($P = 0.009$), favorable site ($P = 0.024$), female gender ($P = 0.034$), T1 stage ($P = 0.034$) and *N*0 stage ($P = 0.037$) were predictive of an improved overall survival. Use of chemotherapy did not affect survival outcome. The patient and tumor characteristics of the 10 patients with embryonal histology are presented in Table 5. Univariate and multivariate analysis for overall survival is presented in Table 6.

The 5-year progression free survival was 21%. On multivariate analysis, female gender ($P = 0.002$), non-embryonal histology ($P = 0.004$), T1 stage ($P = 0.033$) and early IRS Stage ($P = 0.02$) were all associated with an improved progression-free survival.

*Local control and patterns of failure*

The 5-year local control rate was 51%, and multivariate analysis showed that only T1 stage ($P = 0.045$) was predictive for improved local control. Five of six (83%) of Group II patients receiving RT were locally controlled. Among Group III patients, five of eight (63%) receiving RT and one of five (20%) not receiving RT were locally controlled. One of the Group I patients failed in the inguinal nodal region outside of the RT field, which encompassed only the primary site in the thigh. One of three Group I and two of three Group IV patients were locally controlled with postoperative and definitive RT, respectively. Local control according to degree of surgical resection and use of RT is presented in Table 7.

**Table 5.** *Characteristics of patients with embryonal rhabdomyosarcoma*

| | Age range: 16–28 years |
|---|---|
| Gender (male:female) | 8 : 2 |
| Sites | |
| Parameningeal site | 5 |
| Paratesticular site | 3 |
| Retroperitoneum | 1 |
| Orbit | 1 |
| IRS Group | |
| I | 3 |
| II | 1 |
| III | 4 |
| IV | 2 |

IRS, Intergroup Rhabdomyosarcoma Study.

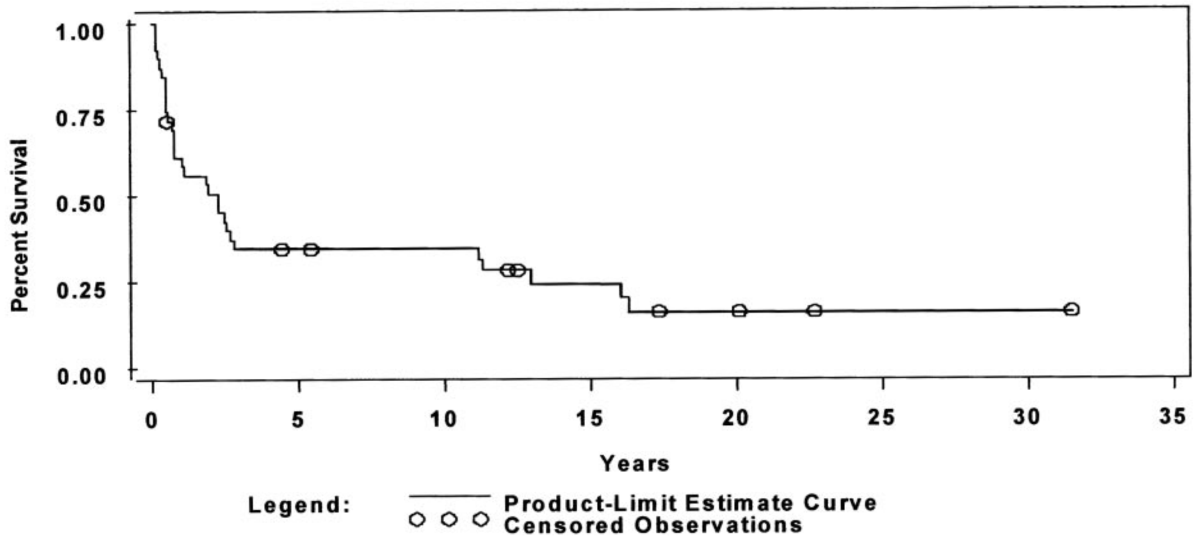

**Fig. 2.** *Overall survival for 39 adult rhabdomyosarcoma cases.*

**Table 6.** *Univariate and multivariate analysis for overall survival*

| Variable | Univariate analysis | Multivariate analysis |
|---|---|---|
| Age | n.s. | n.s. |
| Gender | n.s. | n.s. |
| Time era (pre-1980 vs. post-1980) | n.s. | n.s. |
| T-stage | n.s. | 0.034 |
| N-stage | n.s. | 0.037 |
| M-stage | 0.0046 | n.s. |
| IRS stage | 0.0015 | 0.001 |
| IRS group | 0.0075 | n.s. |
| Histology (E vs. non-E) | n.s. | 0.009 |
| Site | 0.0618 | 0.024 |
| Surgery | n.s. | n.s. |
| Radiotherapy | n.s. | n.s. |
| Chemotherapy | n.s. | n.s. |

n.s., not significant at $P = 0.05$ level; E, embryonal histology.

**Table 7.** *Local control according to degree of surgical resection and use of radiotherapy*

| | Radiotherapy | No radiotherapy | Total |
|---|---|---|---|
| Group I | 1/3 (33%) | 8/11 (73%) | 9/14 (64%) |
| Group II | 5/6 (83%) | – | 5/6 (83%) |
| Group III | 5/8 (63%) | 1/5 (20%) | 6/13 (46%) |
| Group IV | 2/3 (67%) | 1/3 (33%) | 3/6 (50%) |
| Total | 13/20 (65%) | 10/19 (53%) | 23/39 (59%) |

Nine patients did not have any recurrence. Of the remaining 30 patients, nine (23%) had a local recurrence alone, 14 (36%) had distant metastasis alone and seven (18%) had both local and distant metastases. Three of the seven who had local and distant disease also had regional nodal relapse. A total of 16 local failures (33%) occurred at a median time of 4.5 months, all within 54 months from initial diagnosis. A total of 21 cases (54%) had distant spread at a median time of 6 months. Of the three patients who failed at the regional nodes, two had inguinal node recurrence with a lower extremity primary site while another had paraaortic node involvement and a paratesticular tumor.

### Discussion

Rhabdomyosarcoma is the most common soft tissue sarcoma in children accounting for approximately 400 cases per year in the United States. The median age at diagnosis is 5 years and over 80% of cases in children occur before age 15. In the pediatric population, the tumor most commonly arises from the head and neck (40%) followed by the genitourinary sites (20%) and extremities (20%). Embryonal histology and sarcoma botryoides occur in 75% of cases. Pleomorphic histology is almost always not seen. The IRS studies have clearly defined prognostic factors as well as effective chemotherapy agents and RT regimens for this disease. Orbital, paratesticular and non-parameningeal head and neck sites have been found to be favorable location while the parameningeal and extremity sites are considered non-favorable sites. Embryonal histology and sarcoma botryoides have a better prognosis compared to their alveolar counterpart. The success of the multimodality approach to the curative treatment of childhood rhabdomyosarcoma is one of the greatest achievements in the past 35 years. The 3-year survival of 85% in the IRS-IV is a remarkable testimony to the recent advances in the management of this disease.

Adult rhabdomyosarcoma, on the other hand, is less common and, as a result, less information is available to guide clinicians to the treatment and prognosis of the patient. In the most recently reported IRS-IV, 17% of patients were $\geq 16$ years of age. In our study 30% of patients presenting with rhabdomyosarcoma were aged 16 years or greater. Two age peaks were found, one in early adulthood and the other at late adulthood. Although in children treated in IRS-IV, age $\geq 10$ years had a better 3-year failure-free survival than those $> 10$ years (83 vs. 68%, $P = 0.001$), our study showed that age in the adult years did not further classify outcome.

In our study, the extremity was the most common location followed by the head and neck region. Orbital tumor was rare and was seen in only one patient. Pleomorphic histology was the most common subtype; this histological subtype was not found in any of the patients aged 16–19, but was found in 27 and 60% of those aged 20–49 and $\geq 50$ years old, respectively. The Memorial experience also demonstrated the preponderance of pleomorphic histology particularly in adults $> 40$ years of age.[2] Some have questioned the existence of the pleomorphic histology in adults, although recent data confirm its status as a diagnostic entity.[6–8] More commonly in the past, there has been some confusion with the diagnosis of pleomorphic RMS and malignant fibrous histiocytoma (MFH). In fact, two of the pleomorphic rhabdomyosarcomas in our study were originally thought to be MFH, but further immunohistochemical testing supported a RMS diagnosis. Whereas in children, alveolar histology has a worse survival compared to embryonal, our review showed that adults with embryonal histology had the worst survival. One should remember, however, that we had small patient numbers and that five patients treated in the earlier part of the study had NOS histology and could not be further classified. Furthermore, of the 10 patients with embryonal histology, two were Group IV and six had a parameningeal or retroperitoneal tumor. The finding of a worse survival in embryonal histology should be interpreted with caution because of the above reasons. In adults, embryonal histology has not been found to have a better prognosis like in children.[2,9]

The survival of patients with RMS in our series was only 35% at 5 and 10 years and is comparable to the Memorial Sloan-Kettering and Harvard series. Hawkins *et al.* reported the Memorial experience of 84 adult RMS patients and found a similar 5-year survival of 35%.[2] Unlike our series, he found that younger adult patients (aged 16–19) had a better survival compared to those aged $\geq 20$ years. Esnaola *et al.* reviewed 39 adult RMS patients with a 5- and 10-year survival rate of 31 and 27%; age within the adult years, like our study, did not predict for a better outcome.[9]

The survival rates, tumor and treatment characteristics in different and present series are presented in Table 8.[2,9,10] The median age in our series is older compared to the three other reported institutional reviews and is consistent with a higher proportion of pleomorphic RMS. Nodal metastases at initial presentation were seen in 39 of the 178 (22%) accumulated cases, which is in agreement with the pediatric literature and the belief that RMS has a relatively high incidence of lymph node metastasis. Another observation is that almost all patients in the Memorial, Harvard and Stanford reports have received chemotherapy. Despite the consistent use of systemic chemotherapy, the survival results were dismal. We did not find the type of chemotherapy agents used in our study to be beneficial in the overall survival outcome. More effective chemotherapy is needed in the treatment of adults with RMS since 21 of 39 (54%) failed distantly. Because of the high rate of distant metastasis, chemotherapy should

**Table 8.** *Patient, tumor, treatment characteristics and survival rates in contemporary series of adult rhabdomyosarcoma*

| | Memorial (2) $n=84$ | Harvard (6) $n=39$ | Stanford (7) $n=16$ | Iowa (present series) $n=39$ |
|---|---|---|---|---|
| Median age (years) | 23 | 26 | 25 | 45 |
| Age range (years) | 16–76 | 16–82 | 21–67 | 16–80 |
| Histology | | | | |
| Embryonal (%) | 51 | 18 | 56 | 26 |
| Botryoides (%) | 2 | 0 | 0 | 2 |
| Pleomorphic (%) | 17 | 13 | 0 | 36 |
| Alveolar (%) | 30 | 56 | 44 | 21 |
| Mixed (%) | 0 | 0 | 0 | 2 |
| NOS (%) | 0 | 13 | 0 | 13 |
| Primary Site | | | | |
| Extremity (%) | 22 | 31 | 12 | 39 |
| Head and neck (%) | 24 | 33 | 44 | 28 |
| Genitourinary (%) | 21 | 18 | 38 | 20 |
| Other (%) | 33 | 18 | 6 | 13 |
| Tumor Size | | | | |
| $\leq 5$ cm (%) | 36[a] | 38 | na | 33 |
| $> 5$ cm (%) | 43 | 62 | na | 67 |
| % Nodal metastasis[b] | 8 | 46 | 44 | 18 |
| % Distant metastasis[b] | 44 | 33 | 38 | 15 |
| Treatment | | | | |
| Surgery (%) | 75 | 54 | 38[c] | 72 |
| Chemotherapy (%) | 93 | 95 | 88 | 56 |
| Radiotherapy (%) | 49 | 69 | 100 | 51 |
| Overall survival | | | | |
| 5 years (%) | 35 | 31 | 22 | 35 |
| 10 years (%) | na | 27 | na | 35 |

a, does not add to 100% because information is missing; b, at initial presentation; c, may be underestimated as subtotal resections were not included; na, not available.

be strongly considered in the management of adult RMS. In children with RMS, all receive chemotherapy as part of their treatment course. We believe that adult RMS has a worse prognosis compared to children based on our and previously cited institutional reports. The extent of surgical resection was not found to impact on overall survival or local control in our study or the Harvard experience. However, in the Memorial experience, patients with negative margins of resection had a better disease-specific survival compared to those with positive margins of resection. Only tumor invasiveness (T stage) was found on multivariate analysis to predict for local control. The impact of RT on improvement of local control is suggested by our findings in Group II and III patients. Five of six Group II and five of eight Group III patients were controlled with RT while only one of five Group III patients were controlled without RT. This finding must be interpreted with caution given the small number of cases. Because of the small patient numbers it is unclear whether RT is important in Group I or IV patients. RT may be useful in certain subsets of patients with completely resected tumors. In childhood RMS, an analysis of the IRS data indicate an improvement in failure-free and overall survival with the addition of RT in Group I alveolar histology.[11] For children with metastatic sarcoma, the use of RT as consolidative treatment has been found in a series from the University of Chicago to improve local control.[12]

One can conclude that local control is important in the curative treatment of adult RMS. In the Memorial experience, all local failures also experienced distant failure either concurrently or after local recurrence. In our experience, local recurrence was accompanied or followed by distant failure in seven of 16 cases (44%). Isolated nodal failure was not found in any of our patients; all three who had lymph node recurrence also had distant metastases at the time of failure, justifying our current approach of not prophylactically irradiating regional nodes without pathological confirmation of cancer.

Our study is a retrospective analysis of adults with RMS treated over a long period of time. It is also important to note that the possible prognostic factors examined were those applicable to children based on trials conducted by the IRS committee. We also had five patients with a NOS classification and it was not possible for all pathology to be reviewed using modern diagnostic methods. Hence the prognostic significance of histology in our study should be interpreted with caution. Despite these limitations, our findings are important, as there is paucity of information regarding this particular soft tissue sarcoma in adults. In conclusion, adult RMS carries a much worse prognosis compared to childhood RMS. Our study, where a lower frequency of patients received chemotherapy, showed the same poor survival as the Harvard, Memorial and Stanford studies, which almost always routinely employed chemotherapy. We have also observed in adults that tumors tend to occur in the unfavorable sites and, like the Memorial series, have a higher proportion of pleomorphic histology in the older patients. Unfortunately, many patients develop metastatic disease and the optimal chemotherapeutic regimen remains to be defined in this unique population.

### References

1. Crist WM, Anderson JR, Meza JL, Fryer C, Raney RB, Ruymann FB, Breneman J, Qualman SJ, Wiener E, Wharam M, Lobe T, Webber B, Maurer HM, Donaldson SS. Intergroup rhabdomyosarcoma study-IV: results for patients with nonmetastatic disease. *J Clin Oncol* 2001; 19: 3091–3102.
2. Hawkins WG, Hoos A, Antonescu CR, Urist MJ, Leung DH, Gold JS, Woodruff JM, Lewis JJ, Brennan MF. Clinicopathologic analysis of patients with adult rhabdomyosarcoma. *Cancer* 2001; 91:794–803.
3. Kaplan EL, Meier P. Nonparametric estimation from incomplete observations. *J Am Stat Assoc* 1958; 53: 457–81.
4. Cox DR. Regression models and life tables (with discussion). *J R Stat Soc B* 1972; 34: 187–220.
5. SAS System for Windows. Version 8.0, 1999. Cary, NC: SAS Institute, Inc.
6. Gaffney EF, Dervan PA, Fletcher CD. Pleomorphic rhabdomyosarcoma in adulthood. Analysis of 11 cases with definition of diagnostic criteria. *Am J Surg Pathol* 1993; 17: 601–609.
7. Tallini G, Parham DM, Dias P, Cordon-Cardo C, Houghton PJ, Rosai J. Myogenic regulatory protein expression in adult soft tissue sarcomas. A sensitive and specific marker of skeletal muscle differentiation. *Am J Pathol* 1994; 144: 693–701.
8. Wesche WA, Fletcher CD, Dias P, Houghton PJ, Parham DM. Immunohistochemistry of MyoD1 in adult pleomorphic soft tissue sarcomas. *Am J Surg Pathol* 1995; 19: 261–9.
9. Esnaola NF, Rubin BP, Baldini EH, Vasudevan N, Demetri GD, Fletcher CD, Singer S. Response to chemotherapy and predictors of survival in adult rhabdomyosarcoma. *Ann Surg* 2001; 234: 215–23.
10. Prestidge BR, Donaldson SS. Treatment results among adults with childhood tumors: a 20-year experience. *Int J Radiat Oncol Biol Phys* 1989; 17: 507–14.
11. Wolden SL, Anderson JR, Crist WM, Breneman JC, Wharam MD Jr, Wiener ES, Qualman SJ, Donaldson SS. Indications for radiotherapy and chemotherapy after complete resection in rhabdomyosarcoma: a report from the Intergroup Rhabdomyosarcoma Studies I to III. *J Clin Oncol* 1999; 17: 3468–75.
12. Czyzewski EAD, Goldman S, Mundt AJ, Nachman J, Rubin C, Hallahan DE. Radiation therapy for consolidation of metastatic or recurrent sarcomas in children treated with intensive chemotherapy and stem cell rescue: a feasibility study. *Int J Radiat Oncol Biol Phys* 1999; 44: 569–77.
